# Forty years of investment in HIV research: progress towards ending the HIV pandemic and preparation for future pandemics

**DOI:** 10.1002/jia2.26039

**Published:** 2022-11-30

**Authors:** Sarah W. Read, Peter Kim, Mary Marovich, Carl W. Dieffenbach, Anthony S. Fauci

**Affiliations:** ^1^ Division of AIDS National Institute of Allergy and Infectious Diseases National Institutes of Health Bethesda Maryland USA; ^2^ Office of the Director National Institute of Allergy and Infectious Diseases National Institutes of Health Bethesda Maryland USA

1

Since the beginning of the HIV pandemic, sustained and substantial investment in biomedical research and community engagement has led to extraordinary progress towards ending the HIV pandemic and laid a foundation for a successful response to emerging pandemics. This year, on World AIDS Day, we review achievements to date and examine how investments in HIV research were leveraged to develop effective treatment and prevention interventions for COVID‐19. Neither pandemic is over and much remains to be accomplished; however, it is clear that building on successes will help bring an end to the HIV and COVID‐19 pandemics and prepare us to respond to future pandemics.

From the earliest elucidation of the HIV replication cycle leading to the identification of targets for therapeutic intervention, the number of FDA‐approved antiretroviral (ARV) drugs has grown to more than 30 in eight classes. Effective treatment can be as simple as one pill once a day or two injections every 2 months [1]. Anti‐retroviral therapy (ART) not only restores life expectancy to near normal [2], but also eliminates the risk of HIV transmission when consistently used [3]. For those at risk of HIV infection, either daily oral tenofovir‐based medication with emtricitabine or an injection of cabotegravir every 2 months can serve as effective pre‐exposure prophylaxis [4, 5].

In addition to the small molecule anti‐viral approach, the development of broadly neutralizing antibodies (bNAbs) that target highly conserved epitopes on the HIV Env protein portends new strategies for HIV treatment and prevention [6]. Animal studies [7] and clinical trials have demonstrated the anti‐viral potency of bNAbs and have provided a road map to explore bNAbs as modalities of treatment and prevention [8], as well as a mechanism to probe neutralizing epitopes for use in an HIV vaccine [9]. Using the structure‐based design, stabilized, native envelope trimers, which preserve the conformation of bNAb epitopes, define a set of immunogens for sequential immunization strategies that will be used to drive neutralizing antibody breadth [10]. The use of the newer mRNA vaccine platforms to deliver stabilized trimers will accelerate the iterative evaluation of immunogens.

These remarkable scientific milestones resulted from substantial investments in a multitude of research programmes over four decades. Programmes for virology, immunology, drug and vaccine discovery, clinical observational research and clinical trial networks comprised of hundreds of dedicated investigators and research staff around the globe resulted from a deliberate long‐term strategy to profoundly impact the HIV pandemic. Successes during the research response to HIV validated the concept of team science. These scientific discoveries could not have been achieved with short‐term individual grants alone. Instead, sustained investments in collaborative science and ongoing research enterprises allowed for incremental progress to culminate in significant breakthroughs.

The critical role of community engagement was clearly established in the research response to HIV. A successful effort could only have been achieved through the meaningful involvement of persons with HIV (PWH) in research, from planning to implementation. From the early days of the HIV pandemic when advocates stormed the US National Institutes of Health (NIH) to make their priorities known, stakeholder engagement to drive the research agenda has been a cornerstone of the HIV research response. The HIV research enterprise is much improved by the broad involvement of community members on research teams and advisory boards—not only in the United States, but globally, through networks that were established to foster research and care in regions and populations most affected by HIV.

These enduring partnerships, research advances and the availability of ART provided the basis for the President's Emergency Plan For AIDS Relief (PEPFAR). The rapid implementation of research advances to benefit PWH in low‐ and middle‐income countries bolstered global cooperation in the fight against HIV. PEPFAR, the Global Fund and other committed organizations provided the knowledge and resources to advance HIV care and treatment, and fostered implementation and operational science enhancing and strengthening the delivery of high‐quality, cost‐effective care. Expanded access to generic ARVs and molecular diagnostics for the detection of drug resistance and HIV viral load, as well as the adoption of clinical strategies, such as task‐shifting, demonstrated the power of capacity building.

More recently, the HIV research enterprise has served as a foundation for rapid advances during the COVID‐19 pandemic. From virology, structural biology and immunology to community engagement, the research response towards COVID‐19 was made possible, in part, by the years of investment in HIV research. Drug discovery strategies originally developed for HIV have informed many of the structure‐based drug discovery efforts against SARS‐CoV‐2, an example of which is the improvement in the design of protease inhibitor molecules. The payoff from investment in HIV research is also evident in how structure‐based design propelled the SARS‐CoV‐2 vaccine field leading to the rapid design and development of COVID vaccines and monoclonal antibodies. The D614 stabilized pre‐fusion trimer spike of SARS‐CoV‐2, which was designed using the technology developed to stabilize HIV envelopes, serves as the basis of four effective COVID vaccines [11]. Additionally, SARS‐CoV‐2‐specific mAbs were screened for binding ability using the stabilized trimer spike, leading to effective mAb treatments for COVID‐19 early in the pandemic as well as the only agent authorized for use as preexposure prophylaxis [12].

In the clinical sphere, the research infrastructure built to help curtail the HIV epidemic has been successfully leveraged to address the COVID‐19 epidemic. Clinical and research laboratories built for HIV were successfully repurposed to diagnose COVID and identify emerging variants of SARS‐CoV‐2, such as the Omicron variant. NIH‐funded HIV clinical trial networks (ACTG, HPTN, HVTN and INSIGHT) and clinical research sites were engaged to implement phase III vaccine trials as well as phase II/III trials of drugs and anti‐SARS‐CoV‐2 monoclonal antibodies for treatment and prevention. These networks quickly pivoted from HIV to COVID‐19 while engaging the expertise of network scientists to expeditiously implement an extraordinary number of trials. Experienced network laboratory scientists repurposed an HIV‐neutralizing antibody assay to a COVID‐neutralizing assay, which helped to define correlates of protection across all five vaccine efficacy trials [13, 14]. Clinical trialists with HIV experience were consulted by drug companies to inform optimal COVID‐19 preventive trial designs. All of this was done while engaging with affected communities to ensure the appropriate populations were being enrolled and their needs addressed [15].

Looking to the future, we must maintain our focus on ending the HIV pandemic by developing safe, durable HIV vaccines and cures, as well as addressing the non‐infectious comorbidities seen in PWH. This means maintaining long‐term, steady investments in science and infrastructure. Simultaneously, we must retain the ability to quickly pivot and form research collaborations when needed to address public health emergencies. The rapid engagement of the HIV research enterprise, including its community engagement programmes, was essential to the overall agility of the COVID‐19 response. As the most important questions and challenges in HIV research continue to evolve, so must the investments be maintained. These investments will pay off in progress against HIV and will also ensure the flexibility to rapidly respond to future pandemics.

## COMPETING INTERESTS

None of the authors has competing interests.

## AUTHORS’ CONTRIBUTIONS

All five authors contributed to the framing of the Viewpoint. The first four authors, SWR, PK, MM and CWD, drafted the Viewpoint. SWR, CWD and ASF edited the Viewpoint.

## FUNDING

No external support was provided for the preparation of the manuscript.
